# Non-thermal atmospheric pressure plasma activates Wnt/β-catenin signaling in dermal papilla cells

**DOI:** 10.1038/s41598-021-95650-y

**Published:** 2021-08-09

**Authors:** Ji-Hye Hwang, Hyun-Young Lee, Kyung Bae Chung, Hae June Lee, Jino Kim, Kiwon Song, Do-Young Kim

**Affiliations:** 1 Department of Dermatology and Cutaneous Biology Research Institute, Yonsei University College of Medicine, 50-1 Yonsei-ro, Seodaemun-gu, Seoul, 03722 Korea; 2 Research and Development Team, Feagle Co., Ltd, Yangsan, Korea; 3 Department of Electrical Engineering, Pusan National University, Pusan, Korea; 4 New Hair Institute, Seoul, Korea; 5 Department of Biochemistry, College of Life Science and Biotechnology, Yonsei University, Seoul, Korea

**Keywords:** Biophysical chemistry, Mechanisms of disease

## Abstract

There is an unmet need for novel, non-pharmacological therapeutics to treat alopecia. Recent studies have shown the potential biological benefits of non-thermal atmospheric pressure plasma (NTAPP), including wound healing, angiogenesis, and the proliferation of stem cells. We hypothesized that NTAPP might have a stimulatory effect on hair growth or regeneration. We designed an NTAPP-generating apparatus which is applicable to in vitro and in vivo experiments. The human dermal papilla (DP) cells, isolated fresh hair follicles, and mouse back skin were exposed with the NTAPP. Biological outcomes were measured using RNA-sequencing, RT-PCR, Western blots, and immunostaining. The NTAPP treatment increased the expression levels of Wnt/β-catenin pathway-related genes (*AMER3, CCND1, LEF1*, and *LRG1*) and proteins (β-catenin, p-GSK3β, and cyclin D1) in human DP cells. In contrast, inhibitors of Wnt/β-catenin signaling, endo-IWR1 and IWP2, attenuated the levels of cyclin D1, p-GSK3β, and β-catenin proteins induced by NTAPP. Furthermore, we observed that NTAPP induced the activation of β-catenin in DP cells of hair follicles and the mRNA levels of target genes of the β-catenin signaling pathway (*CCND1*, *LEF1*, and *TCF4*). NTAPP-treated mice exhibited markedly increased anagen induction, hair growth, and the protein levels of β-catenin, p-GSK3β, p-AKT, and cyclin D1. NTAPP stimulates hair growth via activation of the Wnt/β-catenin signaling pathway in DP cells. These findings collectively suggest that NTAPP may be a potentially safe and non-pharmacological therapeutic intervention for alopecia.

## Introduction

Hair loss can cause significant psychosocial distress for affected patients, and therapeutic options are limited. Practical use of FDA-approved medications for androgenetic alopecia, such as minoxidil and finasteride, has been restricted due to adverse drug events^1,2^. Thus, there is an urgent need for novel, non-pharmacological therapeutics to treat hair loss.

The concept of targeting Wnt signals as a therapeutic approaches for alopecia stemmed from important role of Wnt/β-catenin signaling in hair development and growth^3,4^. In canonical Wnt/β-catenin signaling, the secreted Wnt ligands bind with the Frizzled receptor, which further inactivates glycogen synthase kinase-3β (GSK-3β), which is an enzyme responsible for the ubiquitination-mediated degradation of β-catenin^4,5^. Finally, nuclear translocation of β-catenin activates transcriptional programs involved in cell proliferation both in hair matrix and dermal papilla. Currently, various drugs and herbal products with features promoting hair growth have been identified as potential activators of Wnt/β-catenin signaling^5,6^.

Non-thermal atmospheric pressure plasma (NTAPP), a partially ionized gas that contains electrically charged particles at atmospheric pressure, has emerged as a potential therapeutic device that could be used in various medicine and biology field^7^. Numerous studies have demonstrated the potential biological benefits of NTAPP, including accelerated wound healing^8,9^, enhanced angiogenesis^10^, and activation of adult stem cells^11,12^. These outcomes prompted us to hypothesize that NTAPP might have a stimulatory effect on hair growth or regeneration. Although a recent study using nitrogen-based NTAPP has shown that plasma increased size of hair follicle in animal models^13^, but impacts of NTAPP on hair growth and its cellular signaling events are still a lack of evidence. Here, we report that NTAPP stimulates hair growth via activation of the Wnt/β-catenin signaling pathway in dermal papilla (DP) cells.

## Results

### Gene expression profiling reveals altered transcriptomes related with Wnt/β-catenin signaling pathway by NTAPP exposure in human dermal papilla cells

**Figure 1:**
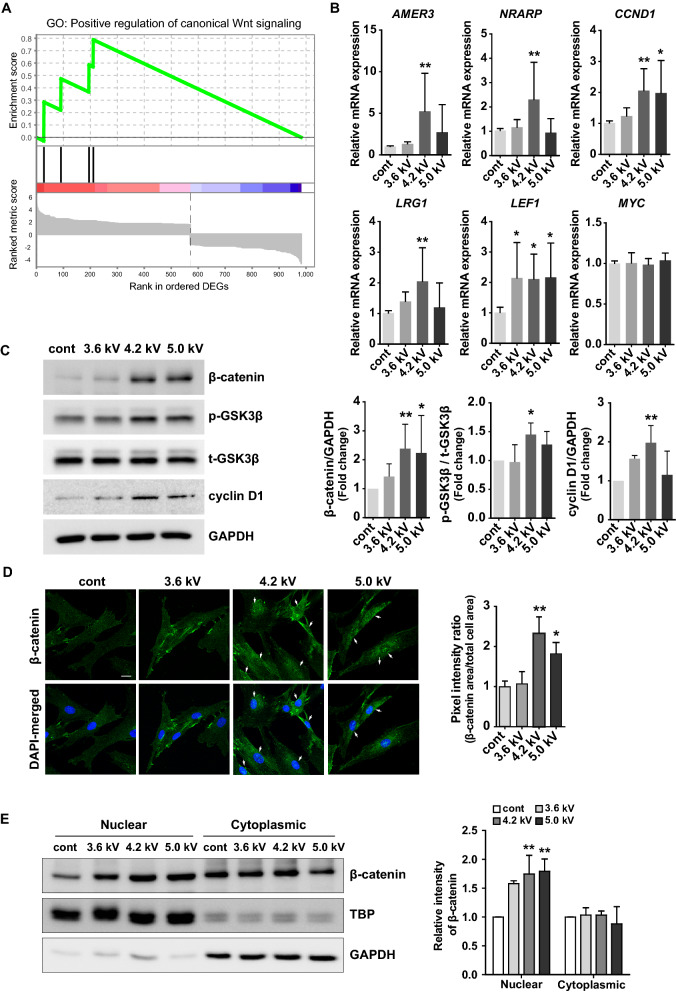
NTAPP activated the Wnt/β-catenin signaling in human dermal papilla (hDP) cells. hDP cells were exposed with NTAPP at 4.2 kV (for RNA-sequencing) or at different plasma doses (3.6 kV, 4.2 kV, and 5.0 kV; for Western blot) for 1 min and were further incubated for 24 h. (**A**) Gene set enrichment analysis for a gene ontology (GO) gene set, GO:0090263. Normalized enrichment score (NES) = 1.812; nominal *p* value = 0.014. A total 983 differentially expressed genes (DEGs) was pre-ranked. (**B**) Quantitative RT-PCR analysis of the expression of selected genes related to the Wnt-β-catenin signaling pathway. Results are expressed as mean ± S.D. of three independent experiments. (**C**) Western blots on β-catenin, GSK3β, and cyclin D1 in hDP cells after NTAPP treatment at different output voltages (3.6, 4.2, and 5.0 kV). (**D**) Immunofluorescent staining of β-catenin in hDP cells treated with NTAPP. Staining intensities of nuclear β-catenin staining were quantified using Image J. Arrows highlight nuclear staining. Scale bar = 20 μm. (**E**) The amounts of β-catenin in both cytoplasmic and nuclear fractions were separately measured. GAPDH and TATA binding protein (TBP) were used for loading controls for cytoplasmic and nuclear fractions, respectively. Results are expressed as mean ± S.D. of three independent experiments. *p* values were determined by one-way ANOVA. *p < 0.05, **p < 0.01 (vs. control). The original images of blots for (**C**, **E**) are available in Supplementary Information.

We designed an NTAPP-generating apparatus using argon as the process gas, which was based on recent approaches^11,12,14^, and applied NTAPP in in vitro and in vivo experiments (details in “Materials and methods” section). The DP, an aggregate of specialized mesenchymal cells at the proximal end of hair follicle, plays a central role in morphogenesis and regeneration of hair follicles to supply inductive signals required for proliferations of matrix keratinocytes^15^. First, we investigated whether NTAPP has a cytotoxic effect by using the 3-(4,5-dimethylthiazol-2-yl)-2, 5-diphenyltetrazolium bromide (MTT) assay at different plasma doses in various exposure time (45, 90, 135, 270 s). We observed that NTAPP had no lethal effect on hDP cells under all tested voltages up to 5.0 kV in all exposure time conditions (Supplementary Figure 1a). Moreover, NTAPP did not induce significant apoptosis which was verified by flow cytometry and quantitative RT-PCR (qRT-PCR) for apoptosis-related genes (Supplementary Figure 1b, 1c). Next, we performed the gene expression profiling using RNA sequencing on NTAPP-treated and control cells. Given the results of MTT assay and Ki-67 staining (Supplementary Figure 2) suggesting an optimal intensity, the human dermal papilla cells were exposed with or without NTAPP (4.2 kV) for 1 min, and were further incubated for 24 h. Based on threshold, log_2_ fold changes > 1.5 or <  − 1.5, 983 genes were differentially regulated. Pathway enrichment analysis on 537 upregulated genes (log_2_ fold changes > 1.5) in NTAPP-exposed hDP revealed that NTAPP has broad biological effects including retinoid metabolism and biological oxidations (Supplementary Figure 3a). Because it is unclear whether biological oxidations and cellular metabolism program directly affect hair cells using this analysis approach, we performed a gene set enrichment analysis (GSEA) using 983 differentially expressed genes (DEGs), which reveals a list of DEGs are enriched in gene ontologies related with appendage development (Supplementary Figure 3b) or positive regulation of canonical Wnt signaling (Fig. 1A). A supervised gene ontology analysis using selected gene sets related with main signaling pathways of hair regeneration and growth (i.e., Wnt-, Notch-, bone morphogenetic proteins (BMP)-, and sonic hedgehog (Shh)-signaling)^3,16^ to identify NTAPP-induced programs related to hair. Indeed, NTAPP induced more transcriptomic changes in genes associated with the Wnt signaling pathway than other pathways include Bmp-, Shh-, and Notch-pathway (Supplementary Figure 3c). Consistently, NTAPP increased genes activating Wnt signaling pathway, which is involved in *AMER3*^17^, *FRZB*^18^, *RSPO2*^19^, *LRP5L*^20^, *GRK5*^21^, *WNT11*^22,23^ and *TMEM198*^24^ (Supplementary Figure 3d). These observations in RNA-seq data were further validated by qRT-PCR for selected Wnt/β-catenin pathway-related genes including *AMER3*, *NRARP*, *CCND1*, *LRG1*, and *LEF1* in hDP cells exposed with various doses of NTAPP (3.6 kV, 4.2 kV, and 5.0 kV) (Fig. 1B). Specifically, we observed that gene expression level of *AMER*3, *NRARP*, *CCND1*, *LRG1*, and *LEF1* were increased 5.2-fold, 2.6-fold, 2.1-fold, 2.2-fold, and 2-fold, respectively, at output voltage of 4.2 kV, whereas only *CCND1* and *LEF1* were increased 2-fold, each, at output voltage of 5.0 kV.

### NTAPP stimulates protein expression of the Wnt/β-catenin signaling pathway in hDP cells

We next measured the levels of proteins related to the Wnt/β-catenin signaling in NTAPP-exposed hDP cells to confirm the findings shown in gene expression profiles. The expressions of β-catenin, p-GSK3β, and cyclin D1 were higher in hDP cells treated with NTAPP, by 2.4-fold, 1.5-fold, and 2-fold, respectively, at an output voltage of 4.2 kV as compared to control groups (Fig. 1C). Because the nuclear localization of β-catenin is crucial in cellular processes such as proliferation, division, and differentiation^4^, confocal microscopy was utilized to study the subcellular localization of β-catenin in hDP cells treated with NTAPP at various output voltages. Immunofluorescence staining revealed that β-catenin was significantly increased and translocated into the nucleus in hDP cells treated NTAPP at 4.2 kV and 5.0 kV, 2.3-fold and 1.8-fold, respectively (Fig. 1D). Subsequently, we further verified that β-catenin was significantly enriched in the nucleus in NTAPP-exposed hDP cells (Fig. 1E). These results suggest that NTAPP affects nuclear accumulation and stabilization of β-catenin via activation of the β-catenin signaling pathway in hDP cells. Based on these results, we determined the optimal exposure conditions of NTAPP at 4.2 kV in hDP cells at which to perform subsequent experiment.

### Canonical Wnt/β-catenin signaling pathway is activated via increased production of Wnt ligands by NTAPP

**Figure 2:**
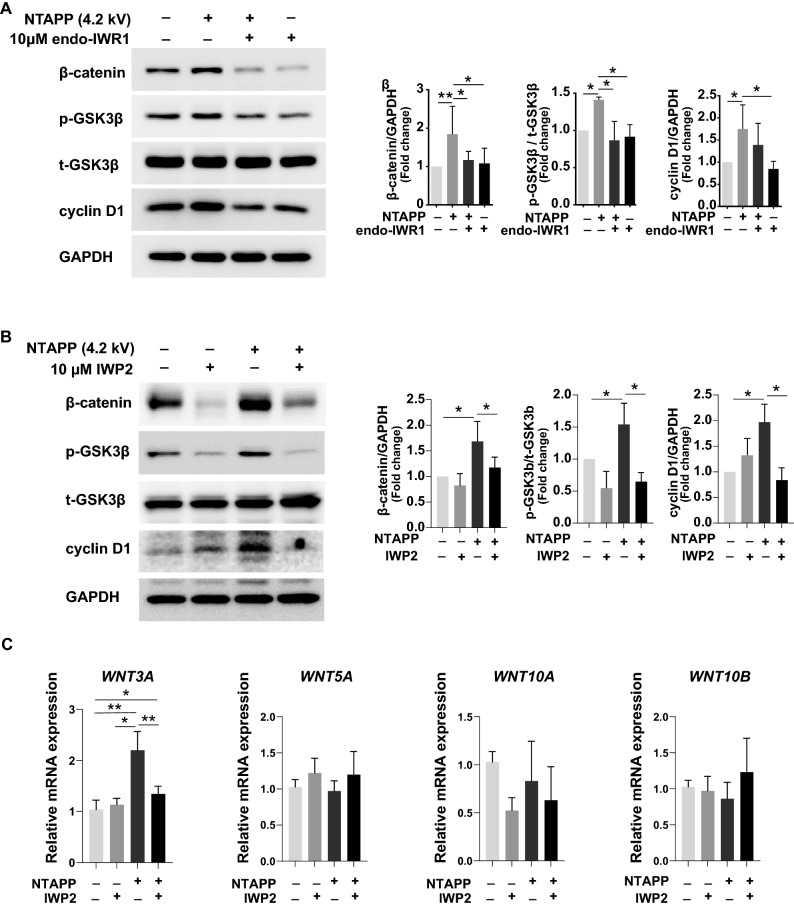
Mode of activation of canonical Wnt/β-catenin signaling by NTAPP. (**A**) Western blots for β-catenin, GSK3β, and cyclin D1 in hDP cells after NTAPP treatment combined with either endo-IWR1 (10 μM) pre-treatment or control. (**B**) Western blots for β-catenin, GSK3β, and cyclin D1 in hDP cells after NTAPP treatment combined with either IWP2 (10 μM) pre-treatment or control. (**C**) qRT-PCR for Wnt ligands after NTAPP treatment with or without pre-treatment of 10 μM IWP2. Cells were treated with NTAPP (4.2 kV) for 1 min, and then incubated for 24 h before harvesting. *p* values were determined by one-way ANOVA. *p < 0.05, **p < 0.01 (vs. control). The original images of blots for (**A**, **B**) are available in Supplementary Information.

To investigate whether NTAPP exposure affect a biological response via the canonical Wnt/β-catenin signaling pathway, we treated hDP cells with two types of pharmacological inhibitor of Wnt/β-catenin signaling, endo-IWR1^25^ and IWP2^26^ before NTAPP treatment. As expected, endo-IWR1, a specific inhibitor of canonical Wnt/β-catenin signaling by increasing degradation of β-catenin^25,26^, substantially reduced the expression of β-catenin in a dose-dependent manner (Supplementary Figure 4a). The western blot analysis revealed that endo-IWR1 significantly abrogated NTAPP-induced expression of β-catenin, p-GSK3β, and cyclin D1 in hDP cells (Fig. 2A), which were also visualized using immunofluorescence staining (Supplementary Figure 4b). Subsequently, we further confirmed that endo-IWR1 suppressed the NTAPP-induced nuclear β-catenin in hDP cells (Supplementary Figure 4c). Similarly, pre-treatment of IWP2, an inhibitor of Wnt secretion by preventing protein palmitoylation^26^, also reduced the NTAPP-induced β-catenin and proteins related downstream signaling (Fig. 2B). Among selected Wnt ligands, the gene expression of Wnt3a significantly induced by NTAPP (Fig. 2C). These results suggest that NTAPP may affect cellular response via the canonical Wnt/β-catenin signaling pathway by stimulating secretion of Wnt ligands in hDP cells.

### NTAPP stimulates the Wnt/β-catenin signaling in human hair organ culture

**Figure 3:**
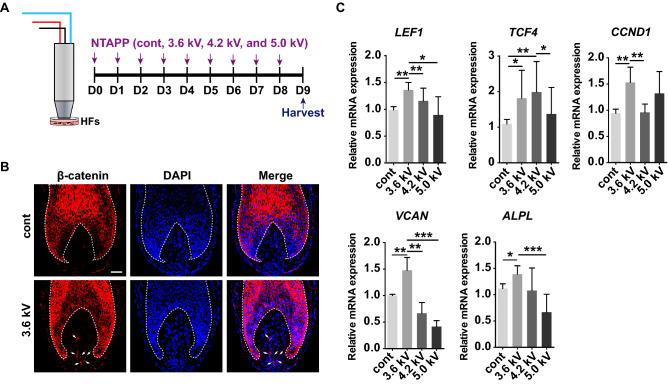
NTAPP activates the Wnt/β-catenin pathway in dermal papilla during hair organ culture. (**A**) Scheme of NTAPP treatment of hair follicles. Isolated human hair follicles (hHFs) were treated with NTAPP (3.6, 4.2, and 5.0 kV) for 1 min on 8 consecutive days. (**B**) Immunofluorescent staining of β-catenin in hHFs. White arrows mark β-catenin staining in dermal papilla (DP). Dotted lines highlight the border between hair keratinocytes and DP cells. Scale bar = 50 μm. (**C**) Quantitative RT-PCR analysis of the selected genes. Results are expressed as mean ± S.D. from five independent experiments (at least 60 follicles in each experiment). *p* values were determined by one-way ANOVA. *p < 0.05, **p < 0.01, ***p < 0.001 (vs. control).

Our data suggest a potential efficacy of NTAPP in preclinical models. Thus, we examined the effect of NTAPP on Wnt/β-catenin signaling pathway activation using an *ex-vivo* human hair follicle (hHF) organ culture model. Freshly isolated hHFs were exposed to 3.6, 4.2, and 5.0 kV of NTAPP for 1 min per day for 8 consecutive days (Fig. 3A). Immunofluorescent staining revealed that NTAPP treatment at a voltage of 3.6 kV induced the activation of β-catenin in DP cells of hHFs (Fig. 3B). We also performed qRT-PCR to quantify the mRNA levels of target genes of the β-catenin signaling pathway (*CCND1*, *LEF1*, and *TCF4*) in hHFs after daily exposure of NTAPP for 8 days. The results showed that NTAPP substantially upregulated the expression levels of *CCND1*, *LEF1*, and *TCF4* as compared with control groups (Fig. 3C). As hair keratinocytes are also major sources of β-catenin, we also verified the increased expression levels of *CTNNB1*, *CCND1, LEF1*, and *TCF4* in mechanically isolated DPs (Supplementary Figure 5). Interestingly, the expression levels of two well-known DP cell markers for hair inductivity, versican (*VCAN*) and alkaline phosphatase (*ALPL*)^27–29^ were also increased after NTAPP treatment (Fig. 3C). These combined results reiterate potentially broad, beneficial effects of NTAPP on hair growth (Supplementary Figure 3a). However, NTAPP did not induce a statistically significant elongation of hHFs in the ex vivo organ culture model (data not shown), and Ki-67+ keratinocytes in matrix were not significantly increased in NTAPP-exposed hHFs (Supplementary Figure 6). Nevertheless, our results suggest that NTAPP potentially modulates Wnt/β-catenin signaling by stimulating hDP cells.

### NTAPP enhances hair regrowth via Wnt/β-catenin signaling pathway in mouse model

**Figure 4:**
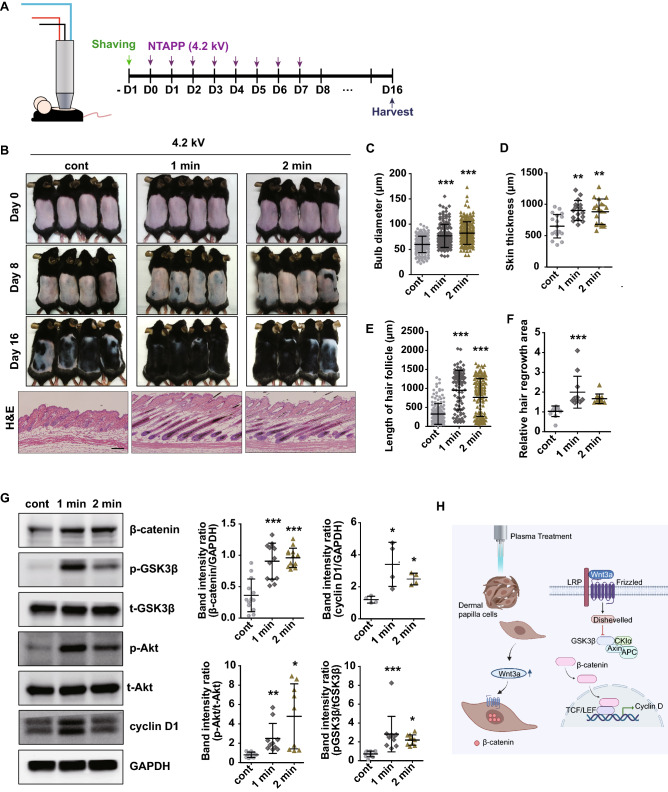
NTAPP stimulates the Wnt/β-catenin pathway and enhances hair regrowth in a mouse model. (**A**) The back skins of 7-week-old female C57BL/6 mice (*n* = 5–6 per group, three independent experiments) were shaved and treated with NTAPP (4.2 kV) for 1 or 2 min on 7 consecutive days. (**B**) Gross images of mouse dorsal skin on day 0, 8 and 16 days after the start of NTAPP treatment (top panels); hematoxylin and eosin (H&E) staining of the mouse back skins (lower panels, scale bar = 200 μm). (**C**–**F**) Histological analysis of bulb diameter, skin thickness, hair follicle length, and relative hair regrowth area. (**G**) Western blot analysis of β-catenin, GSK3β, AKT, and cyclin D1 levels in NTAPP-treated mouse skin. (**H**) A graphical summary of results. All results presented are representative of at least three independent experiments. *p* values were determined by one-way ANOVA. *p < 0.05, **p < 0.01, ***p < 0.001 (vs. control). The original uncropped images for g are available in Supplementary Information.

To determine the in vivo effects of NTAPP, the back skin of 7-week-old female C57BL/6 mice was treated daily with NTAPP at voltage of 4.2 kV for 1 or 2 min at a distance of 0.2 mm from the shaved skin surface for 7 consecutive days (Fig. 4A). At day 16, we observed that NTAPP-treated mouse exhibited markedly increased anagen induction and hair growth much more efficiently than control groups (Fig. 4B). To further verify with more accurate analysis, morphological characteristics of hair growth stage in mice, we evaluated HF length, skin thickness, bulb diameter, and hair regrowth area on digital image of H&E staining at day 16 (Fig. 4C–F). Histologic analyses revealed that the bulb diameter, skin thickness, and length of HF were significantly increased in NTAPP-treated mouse more than control groups (Fig. 4C–E). Moreover, qualitative image analyses showed a prominent increase in hair regrowth area in NTAPP-treated mouse more than control groups (Fig. 4F). These results show that NTAPP could stimulate hair growth in mouse model.

To confirm whether Wnt/β-catenin signaling pathway is involved in NTAPP-induced anagen induction and hair growth, we next measured the levels of protein expression related with Wnt/β-catenin signaling pathway using Western blot analyses (Fig. 4G). We found that NTAPP treatment significantly increased the protein levels of β-catenin, p-GSK3β, p-AKT, and cyclin D1 in the exposed mouse skin more than control groups. Immunohistochemical staining of β-catenin also highlight the expression of β-catenin in dermal papilla of NTAPP-exposed skin (Supplementary Figure 7). These data indicate that NTAPP promotes hair growth in mice via activation of the β-catenin signaling pathway in murine skin (graphical summary in Fig. 4H).

## Discussion

Recently, NTAPP has been studied for its beneficial effects such as proliferation, angiogenesis, maintaining the stem cell characteristics, and promoting wound healing^8–12,30^. Furthermore, therapeutic evidences in pre-clinical models of inflammatory skin disease have been also reported^31–33^. However, the effect of NTAPP on hair growth or regeneration still remains unknown. In this study, we demonstrated that NTAPP could stimulate hair growth by indirectly activating the Wnt/β-catenin pathways at both cellular and tissue levels.

In the first part of this study, NTAPP increased the expression of genes related to the biological oxidation-mediated- and the Wnt/β-catenin- pathways. Previous studies have shown that NTAPP can generate different types of reactive oxygen species^8,10–12,34–37^. The redox signaling can regulate the canonical Wnt/β-catenin signaling pathway by activating TCF^17,38,39^ and that NTAPP impacts on the biological targets by generating the various oxidation molecules^10–12,30,35,40^. With this background, NTAPP-generated free radical species can be putative drivers modulating Wnt/β-catenin signaling pathway in DP cells. However, this direct connection between redox signaling and the Wnt/β-catenin signaling needs to be further evaluated in hair cells.

As expected, the NTAPP-induced nuclear β-catenin was abrogated by treatment of endo-IWR1, an inhibitor of canonical Wnt/β-catenin signaling. Interestingly, NTAPP induced expression of Wnt3a among the tested Wnt ligands. The pre-treatment of IWP2, an inhibitor preventing secretion of Wnt ligands, abrogated Wnt/β-catenin activation. Thus, these observations show that NTAPP increased the secretion of Wnt ligands from DP cells. In the same line, results from *ex-vivo* organ culture model using human hair follicles again showed that NTAPP substantially increased the components of Wnt signal transduction cascade including *CCND1*, *LEF1*, and *TCF4*, and the activation of β-catenin in DP cells. Taken together, these results suggest that NTAPP could be responsible for activating Wnt/β-catenin signaling pathway via inducing production of Wnt ligands in hDP cells. However, the NTAPP exposure did not induce a statistically significant elongation of hHFs in the ex vivo organ culture model. Because we could observe significant numbers of Ki-67+ matrix keratinocytes even in NTAPP-untreated hair bulbs, the effect of NTAPP might be obscured in ex vivo conditions in which exogenous factors stimulating hair growth may exist. Instead, in an animal model, hair shaft elongation was promoted by NTAPP exposure on the mouse back skin, and NTAPP induced the telogen-anagen transition and increased length and thickness of HFs. Induction of Wnt/β-catenin pathway components, a putative mode of action of NTAPP, was commonly observed in both models, the HF organ culture and in vivo mouse skin. A missing part of this work is how the other cellular compartments surrounding DP contribute to hair growth after NTAPP exposure in vivo. Recently, several studies have shown that NTAPP can control biological functions of keratinocytes and stromal cells including fibroblasts^41,42^. Considering complicated reciprocal signaling among various cellular components of HF^16^, a holistic understanding on biological effects of NTAPP in each cellular subset is necessary for further clinical application.

Additional, potential obstacles for the clinical application of NTAPP are the optimal dosing and the penetration depth. We have applied different voltages of NTAPP based on experimental models used, suggesting fine tuning for both optimal fluence and repetitive intervals should be optimized based on clinical applications. In terms of penetration depth, the effect of argon-based plasma treatment could reach up to dermis in our previous observations^14^. In this context, promoting hair growth in mouse skin may not be assure successful application of NTAPP into thicker human skin such as scalp. Therefore, indirect application of NTAPP such as plasma-treated medium can be a feasible approach to overcome the penetration issue^43^. Notably, we have shown the maintenance of hair-inductive ability verified by upregulated *ALPL* and *VCAN* after NTAPP exposure on isolated fresh hHFs, which implies NTAPP can be applied on a novel device enhancing viability or survival rate of grafts during hair transplantation^44^.

In conclusion, our study suggests that NTAPP activates Wnt/β-catenin signaling in DP cells, which is a pivotal cue for hair growth and regeneration. This study focused on the effects of NTAPP in DP cells. Thus, future studies are needed to fully investigate the complex biological impact of NTAPP on the entire hair mini-organ. Nevertheless, our data suggest that NTAPP may be a potentially safe and non-pharmacological therapeutic intervention for hair loss.

## Materials and methods

### Source of human hair follicle and isolation of dermal papilla cells

Occiput scalp containing mainly anagen VI hair follicles was obtained from volunteers. Hair follicles that were morphologically considered to be in anagen were used in this study. Hair follicles were isolated under a stereomicroscope, and DP were separated from individual hair follicles using 31G fine needles as previously described^45,46^. Then DP cells were isolated from the separated DP as previously described^47^. Briefly, approximately 15–20 DP were cultured per a 60 mm dish in 5 ml of Dulbecco's modified Eagle's medium (DMEM; Hyclone, Logan, UT, USA) supplemented with 20% fetal bovine serum (FBS; Hyclone) and antibiotic/antimycotic solution (Gibco BRL, Gaithersburg, MD, USA) containing penicillin and streptomycin at 37 °C in a 5% CO_2_ incubator. When cellular confluency reaches about 80%, DP cells were harvested and stored to 6.5 × 10^5^ cells per a vial with 1 ml of fetal bovine serum (FBS; Hyclone) with 10% DMSO (Sigma, USA) at − 80 °C until used.

### Cell culture and the pre-treatment of inhibitors of Wnt/β-catenin signaling

Human dermal papilla cells (hDP cells) were cultured in Dulbecco's modified Eagle's medium (DMEM; Hyclone, Logan, UT, USA) supplemented with 10% fetal bovine serum (FBS; Hyclone) and antibiotic/antimycotic solution (Gibco BRL, Gaithersburg, MD, USA) containing penicillin and streptomycin. Cells were incubated at 37 °C in a 5% CO_2_ incubator. All cultures used for experiments were in the third passage. In order to expose NTAPP on cells, 6.5 × 10^4^ cells were seeded in a 35-mm culture dish and incubated for 24 h. The cells were exposed to 3.6 kV, 4.2 kV, and 5.0 kV of NTAPP for 1 min, the NTAPP-exposed cells were further incubated for 24 h before harvesting. The distance between the device and cells was fixed to 1 cm, and 1. 5 ml of medium was used.

To understand which step of Wnt/β-catenin signaling is affected by NTAPP, experiments using two inhibitors for Wnt/β-catenin signaling pathway were performed. The hDP cells were pre-treated with or without 10 μM endo-IWR1 or 10 μM IWP2 (both from Tocris Bioscience, Bristol, UK) for 24 h before other experiments.

### Hair follicle organ culture

Briefly, after separation of the hair follicles (HFs) under a binocular dissecting microscope, the proximal two-thirds of anagen HFs located in the subcutaneous fat were isolated using fine forceps (Scitech Korea Inc., Korea), and subsequently collected in a 35-mm culture dish containing complete hair follicle culture medium (Williams E; Gibco BRL) supplemented with 2 mM/l-glutamine (Gibco BRL), 10 ng/ml hydrocortisone (Sigma-Aldrich, St Louis, MI, USA), and 10 μg/ml insulin (Invitrogen, Carlsbad, CA, USA).

Isolated human HFs were cultured in a 35-mm culture dish containing 1.5 ml complete hair follicle culture medium. The culture medium was replaced every 2 days. The human HFs were exposed to 3.6 kV, 4.2 kV, and 5.0 kV of NTAPP for 1 min per day 7 consecutive days. The distance between the device and bottom of culture dish was fixed to 1 cm. Each experiment was repeated with hair follicles harvested from different donors.

### Design of NTAPP device

We designed an NTAPP-generating apparatus using argon as the process gas, which was based on recent approaches^11,12,14^, and applied NTAPP in in vitro and in vivo experiments. The schematics of the experimental setup and NTAPP device are shown in Supplementary Figure 8. NTAPP is the coaxial dielectric barrier discharge type which composed of a dielectric glass tube with an outer diameter of 6.35 mm, grounded cylindrical meshed electrode, and a brass inner electrode inserted in the glass tube. The argon gas flows between a Teflon body and the meshed outer electrode with 5 standard liter per minute (slm) by mass flow controller. The peak-to-peak sinusoidal voltage generated by high voltage circuit was applied to the central brass inner electrode from 0 to 5 kV with frequency of 25 kHz, while the meshed electrode was grounded. Within experimental conditions (3.6–5.0 kV), temperature measured 5 mm from the plasm jet remained below 36 °C.

### NTAPP treatment and cell viability and proliferation assay

After exposure to NTAPP, cell viability, apoptosis and cellular proliferation were evaluated using 3-(4,5-dimethylthiazol-2-yl)-2,5-diphenyl-2H-tetrazolium bromide (MTT) assay, flow cytometry using propidium iodide and annexin V, and Ki-67 staining. The detailed methods and reagents are described in Supplementary Information.

### Western blot analysis

The levels of cellular proteins were quantified using Western blot analysis by utilizing primary antibodies: anti-β-catenin (1:1000), anti-pGSK3β (Ser9, 1:1000), anti- tGSK3β (1:1000), anti-cyclin D1 (1:1000), anti-p-Akt (1:1000), anti-tAkt (1:1000) (all from Cell Signaling Technology, Danvers, MA, USA), or anti-GAPDH (1:5000; Santa Cruz Biotechnology, Dallas, TX, USA). Detailed methods are provided in Supplementary Information.

### Isolation of nuclear and cytoplasmic proteins

Nuclear and cytoplasmic proteins were isolated using the NE-PER Nuclear and Cytoplasmic Extraction Reagents kit following the detailed instructions provided by the manufacturer (Thermo scientific, Rockford, IL, USA). Protease inhibitor solution (GenDEPOT, Katy, TX, USA) was added to cytoplasmic extraction reagent I (CERI; Thermo scientific) and nuclear extraction reagent (NER; Thermo scientific) prior to use.

### Gene expression profiling, data analysis and pathway analysis

Total RNAs from NTAPP-treated cells or non-treated cells were purified and isolated using an RNeasy plus mini kit (Qiagen, Hilden, Germany). RNA quality was assessed by Agilent 2100 Bioanalyzer using the RNA 6000 Nano Chip (Agilent Technologies, Amstelveen, The Netherlands), and RNA quantification was performed using ND-2000 Spectrophotometer (Thermo Inc., DE, USA). Sequencing libraries were prepared using the SMARTer Stranded RNA-Seq Kit (Clontech Laboratories, Inc., USA) and the Poly(A) RNA Selection Kit (LEXOGEN, Inc., Austria) by the manufacturer’s recommendation. Quantification was performed using the library quantification kit using a StepOne Real-Time PCR System (Life Technologies, Inc., USA). High-throughput sequencing was performed as paired-end 100 sequencing using HiSeq2500 (Illumina, Inc., USA). Pathway analysis and gene set enrichment analysis on differentially expressed genes were performed using metascape web tool (metascape.org)^48^ and GSEA (https://www.gsea-msigdb.org/gsea/index.jsp)^49^, and selected annotated gene lists from a gene ontology database (https://www.ebi.ac.uk/QuickGO/) were matched with differentially expressed genes. The data of gene expression profiling have been uploaded to the NCBI Gene Expression Omnibus under accession number GSE152537.

### RNA isolation and quantitative RT-PCR

Total RNA from cells was isolated with Trizol reagent (Qiagen GmbH, Hilden, Germany) and RT-PCR was conducted using a quantitative SYBR Green RT-PCR kit (Applied Biosystems, Warrington, UK) was used with a Step One Plus RT-PCR System (Applied Biosystems). The list of primers used during experiments is shown in Supplementary Table 1. Detailed methods are provided in Supplementary Information.

### Immunofluorescence

The 6.5 × 10^4^ hDP cells were transferred into a 35-mm confocal dishes (SPL life sciences, Korea) and then exposed to NTAPP 24 h after seeding. The NTAPP-exposed hair follicles were embedded in OCT compound (Tissue Tek, Torranc, CA, USA) at − 80 °C and then sectioned to 5 μm at − 20 °C. The cells or tissue sections were fixed in 4% paraformaldehyde in PBS for 15 min, and permeabilized with 0.1% Triton X-100 (Sigma-Aldrich) and blocked with 5% goat serum in PBS and then incubated with anti-β-catenin (1:100; Cell Signaling technology, Beverly, MA, USA) for overnight at 4 °C. After washing with PBS, samples were incubated with an Alexa Fluor 488 goat anti-rabbit IgG (Life Technologies, Eugene, OR, USA). Finally, samples were washed with PBS and counterstained with 4,6-diamidino-2-phenylindole (DAPI) (Life Technologies). The images were acquired using a Zeiss LSM 700 confocal laser scanning microscopy software (Carl Zeiss, Oberkochen, Germany). For quantitative analyses, ratio of cells positive for nuclear β-catenin staining to total number of cells was counted as pixel intensity ratio positive for nuclear localization using Image J (NIH, Bethesda, MD, USA).

### Animal studies

The animal study was approved by the Institutional Animal Care and Use Committee (IACUC) at Yonsei University (Seoul, Korea) under IACUC Approval No. 2015-0254. All methods regarding animal use were carried out in accordance with relevant guidelines and regulations. The study was carried out in compliance with the ARRIVE guidelines.

The back skins of 7-weeks-old C57BL/6 mice (Japan SLC, Inc. Shizuoka, Japan) were shaved then depilated using a depilatory cream (Veet, Reckitt Benckiser, Cedex, France). The shaved skin was exposed to 4.2 kV NTAPP for 1 min or 2 min daily up to 7 days.

### Statistical analysis

All data are obtained as mean ± SD for parametric data, and as median and interquartile range for nonparametric data. The GraphPad Prism, version 8.4.3 (GraphPad Software, Inc., San Diego, CA, USA) was used to assess the significance of differences between two groups by Student’s t-test or a one-way ANOVA with a Bonferroni test. A *p* value < 0.05 was considered statistically significant.

### Ethics approval and consent to participate

This study was approved by the Institutional Review Board at Severance Hospital, Seoul, Korea (4-2014-0830) and all sample donors provided written informed consent. All experimental procedures using human materials were conducted according to the Declaration of Helsinki Principles.

### Consent for publication

Authors declare that we all participated in the study and the development of the manuscript. All authors give our consent for the article to be published in Scientific Reports.

## Supplementary Information

Supplementary Information.

## Data Availability

Data are available from the authors upon reasonable request. Gene expression matrices for RNA-sequencing are available in GEO depository; https://www.ncbi.nlm.nih.gov/geo/query/acc.cgi?acc=GSE152537.

## Abbreviations

NTAPP: Non-thermal atmospheric pressure plasma
DP: Dermal papilla
hDP: Human dermal papilla
HF: Hair follicle
