# Supplementary material for: Non-thermal atmospheric pressure plasma activates Wnt/β-catenin signaling in dermal papilla cells
Source: Sci Rep. 2021 Aug 9;11:16125. doi: 10.1038/s41598-021-95650-y (PMC8352944; doi:10.1038/s41598-021-95650-y)
Supplement: Supplementary file 1 — Supplementary Information. [file 41598_2021_95650_MOESM1_ESM.pdf]

Non-thermal atmospheric pressure plasma activates Wnt/β-catenin signaling in dermal papilla cells

Ji-Hye Hwang[1], Hyun-Young Lee[2], Kyung Bea Chung[1], Hae June Lee[3], Jino Kim[4], Kiwon Song[5] and Do-Young Kim[1*]

List of Supplementary Materials

Supplementary Figure Legends

Supplementary Figure 1. NTAPP has no toxic effect on human dermal papilla (hDP) cells.
Supplementary Figure 2. Ki-67 staining in NTAPP-treated hDP cells.
Supplementary Figure 3. NTAPP activated the Wnt/β-catenin signaling pathway-related genes in hDP cells.
Supplementary Figure 4. Endo-IWR1 attenuates NTAPP-induced β-catenin signaling pathway in hDP cells.
Supplementary Figure 5. NTAPP upregulated the expressions of *LEF1*, *TCF4*, *CCND1,* and *CTNNB1* in mechanically isolated dermal papilla.
Supplementary Figure 6. Changes in Ki-67+ hair matrix cells after NTAPP exposure in hair organ culture model.
Supplementary Figure 7. Expression of β-catenin in dermal papilla after NTAPP exposure in animal model.
Supplementary Figure 8. Scheme of a customized NTAPP-generating device.

Supplementary Table 1. The list of RT-PCR primers used in this study

Full-length gels and blots for cropped images

Original gels/blots for Figure 1c, e
Original gels/blots for Figure 2a, b.
Original gels/blots for Figure 4g.
Original gels/blots for Supplementary Figure 4a,c

Supplementary Method

Cell viability, apoptosis, and proliferation assay
Western blot analysis
RNA isolation and quantitative RT-PCR
Immunohistochemical staining

Supplementary Figure 1. NTAPP has no toxic effect on hDP cells.

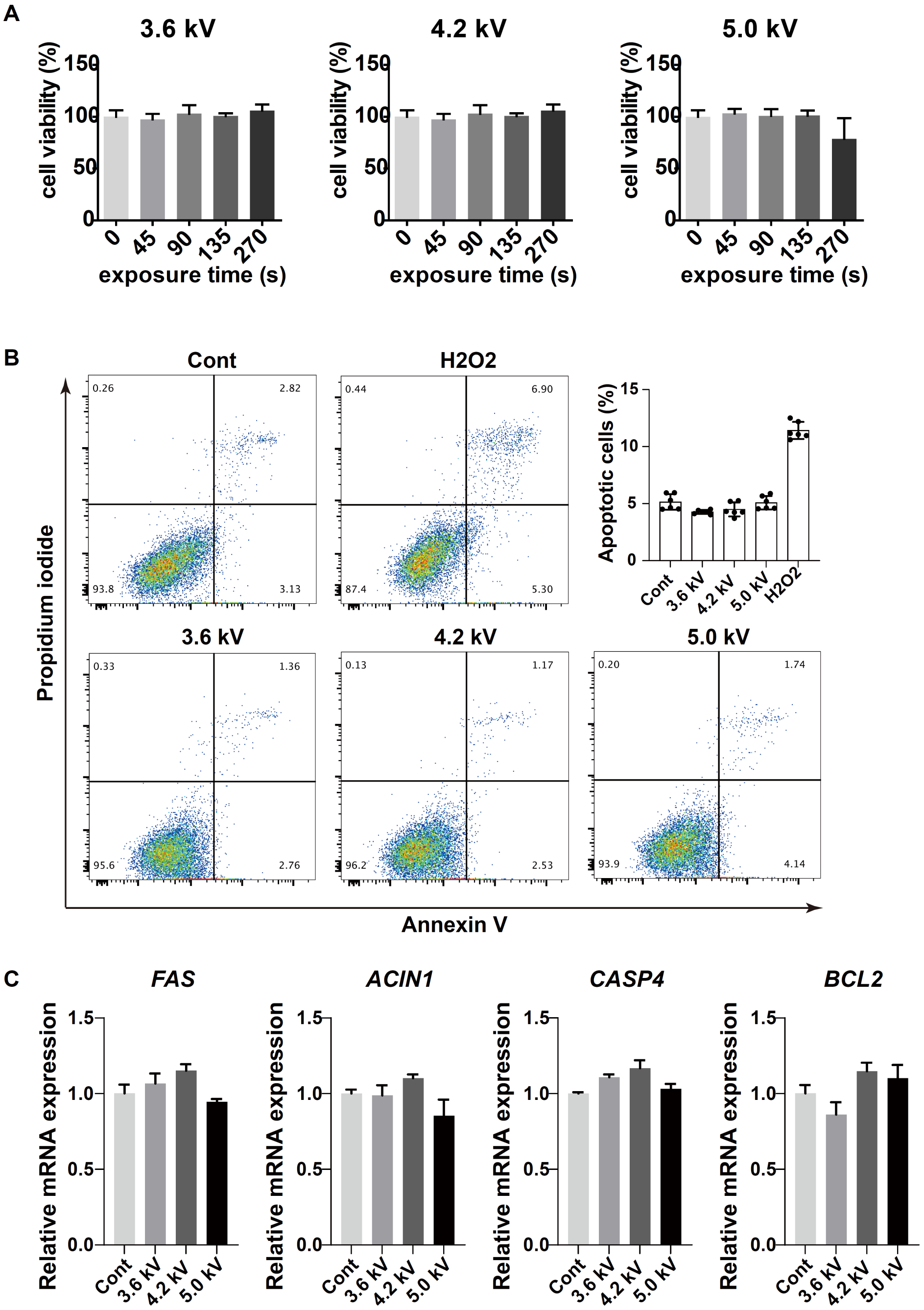

(A) hDP cells were exposed with several treatment times (45, 90, 135, and 270 s) of NTAPP at different plasma doses (3.6 kV, 4.2 kV, and 5.0 kV) and were further incubation for 24 h from the initial exposure. Assays were repeated in triplicate. (B) Flow cytometry analysis for apoptosis of hDP cells after NTAPP exposure. Cells were stained using propiodium iodide and annexin V. A representative images of flow cytometry from multiple replicates (n=6). All annexin V+ cells were measured as apoptotic cells (both early and late). Hydrogen oxide (H2O2) was used as positive control for apoptosis. (C) Quantitative RT-PCR on apoptosis related genes. Multiple comparisons were performed by one-way ANOVA. Values are means ± S.D.

Supplementary Figure 2. Ki-67 staining in NTAPP treated hDP cells.

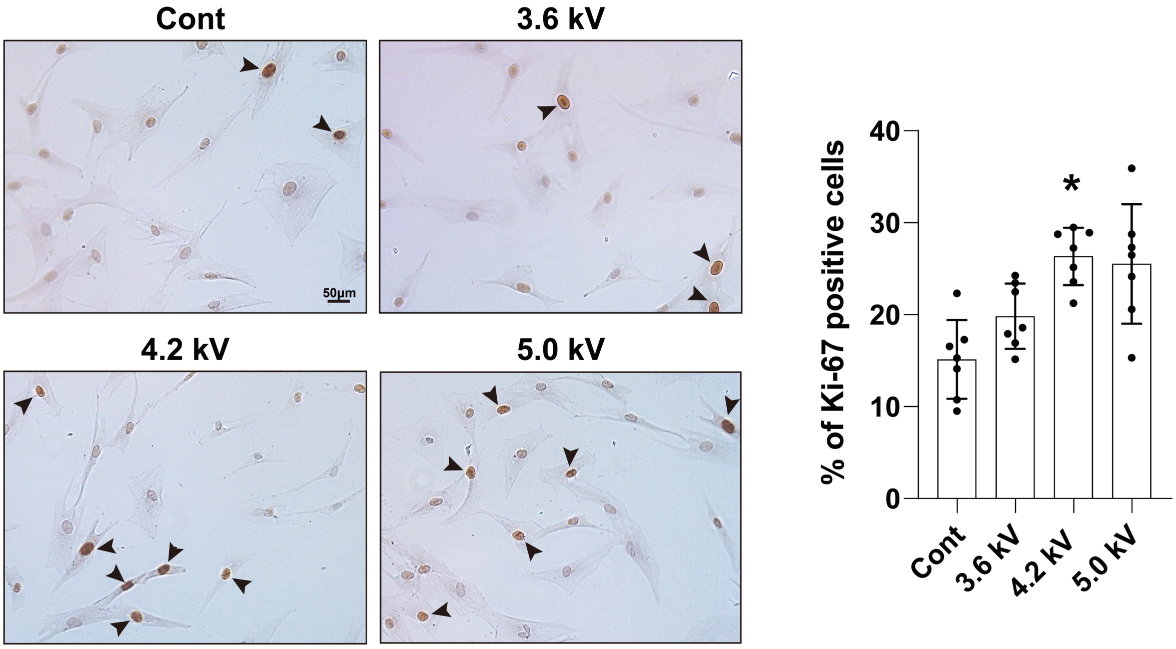

Ki-67 staining in NTAPP treated hDP cells. The hDP cells were exposed with NTAPP for 60 s at different plasma doses (3.6 kV, 4.2 kV, and 5.0 kV) and were further incubation for 24 h from the initial exposure. Cells with nucleus stained intensely against Ki-67 were counted by two independent observers (J.H.H., D.Y.K.). Arrowheads indicate positive staining of Ki-67. Multiple comparisons were performed by one-way ANOVA (n=7 replicates for each group). *$P<0.05$.

Supplementary Figure 3. NTAPP activated the Wnt/β-catenin signaling pathway-related genes in hDP cells.

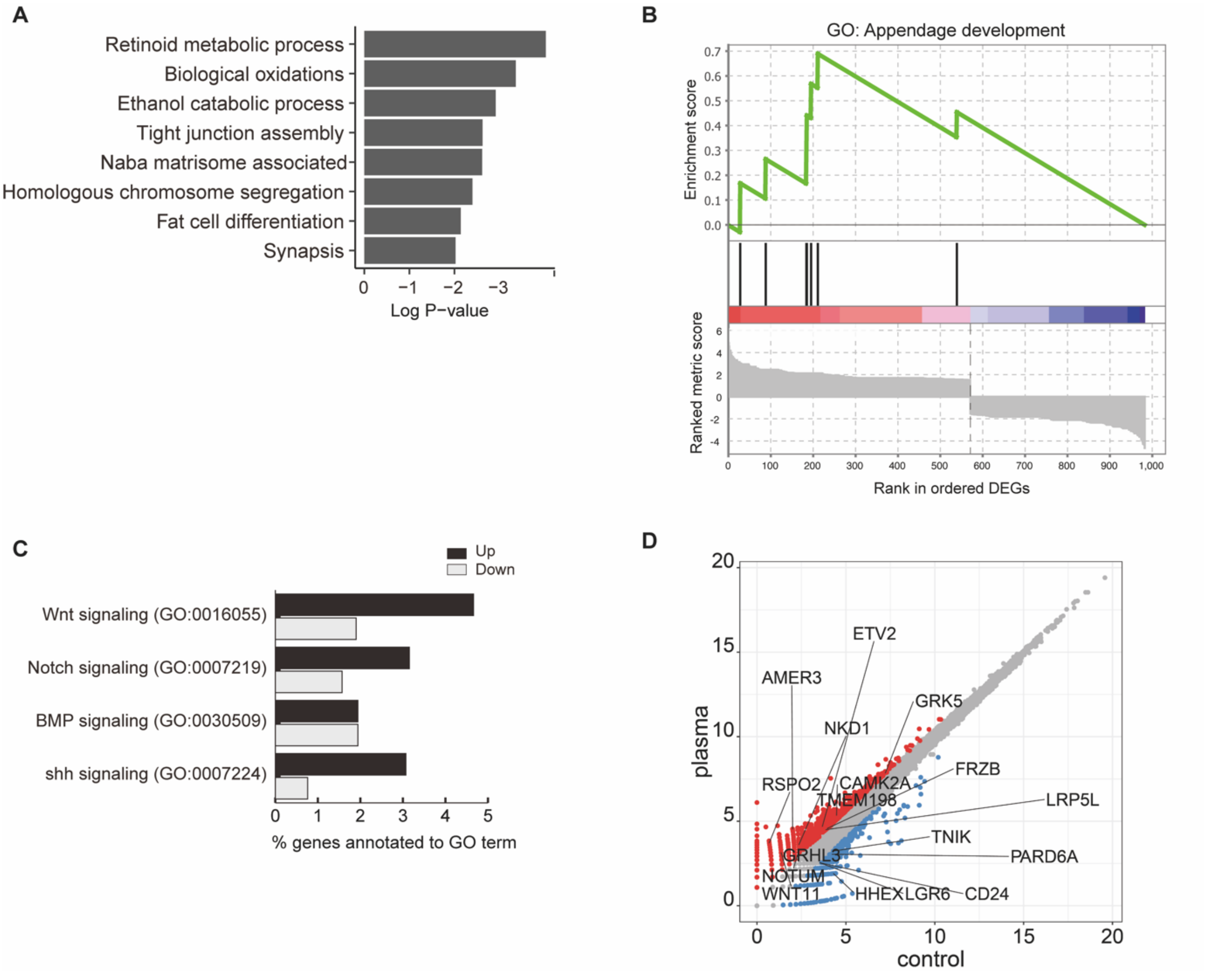

The gene expression profiling was performed using RNA sequencing on NTAPP-treated and control cells. The human dermal papilla cells were exposed with (n=1) or without (n=1) NTAPP (4.2 kV) for 1 min, and were further incubated for 24 h. Based on threshold, log_2 fold changes >1.5 or <-1.5, 983 genes were differentially regulated.

(A) Metascape analysis based on pathway using 537 upregulated genes in NTAPP exposed hDP cells (vs. control). All differentially regulated genes with log_2 fold change > 1.5 were included. (B) Gene set enrichment analysis for a gene ontology (GO) gene set, GO:0048736. Normalized enrichment score (NES)=2.086; nominal p-value<0.001. A total 983 differentially expressed genes (DEGs) was pre-ranked. (C) Differentially expressed genes from RNA sequencing of NTAPP-treated hDP cells were matched with the gene sets of selected GO terms. (D) Selected differentially expressed genes related with Wnt signaling pathway were shown in a scatter plot. Values mean log_2(read count+1). Up-regulated (red) and down-regulated (blue) genes with fold change > 1.5 or <0.5, respectively.

Supplementary Figure 4. Endo-IWR1 attenuates NTAPP-induced β-catenin signaling pathway in hDP cells.

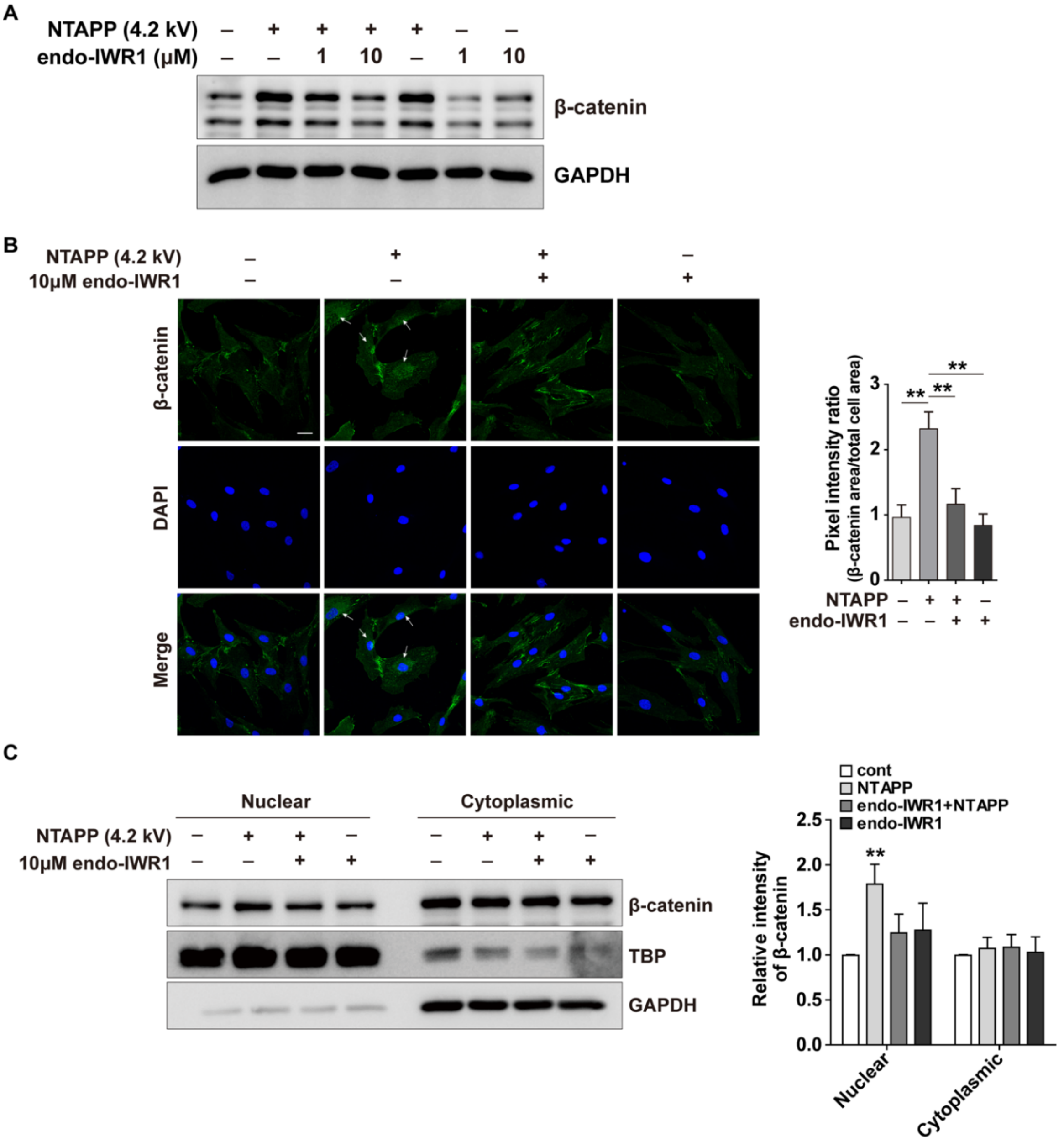

(A) Endo-IWR1 inhibits NTAPP-induced β-catenin in a dose-dependent manner in hDP cells. hDP cells were pre-treated with endo-IWR1 (1 or 10 μM) or DMSO for 24 h, and then exposed with NTAPP (4.2 kV) for 1 min, and were further incubated for 24 h. Western blot for β-catenin were measured. GAPDH served as a loading control. The result is a representative from three independent experiments. (B) Immunofluorescent staining of β-catenin in hDP cells treated with NTAPP combined with endo-IWR1 pre-treatment. Staining intensities of nuclear β-catenin staining were quantified using Image J. Arrows highlight nuclear staining. Scale bar = 20 μm. (C) The levels of β-catenin were measured in both cytoplasmic and nuclear fractions. GAPDH and TATA binding protein (TBP) were used for loading controls for cytoplasmic and nuclear fractions, respectively. Results are expressed as mean ± S.D. of three independent experiments. P-values were determined by one-way ANOVA. **p< 0.01 (vs. control).

Supplementary Figure 5. NTAPP upregulated the expressions of *LEF1*, *TCF4*, and *CCND1* in mechanically isolated dermal papilla.

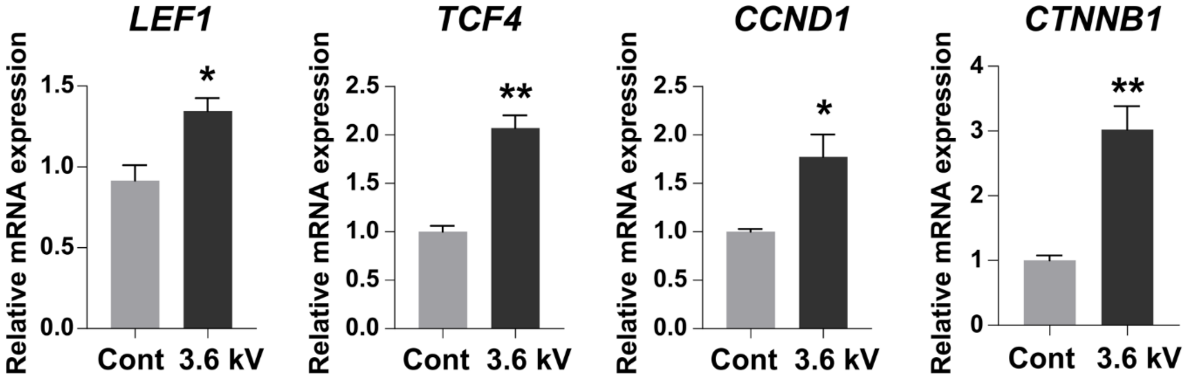

Isolated human hair follicles (HFs) were cultured in a 35-mm culture dish containing 1.5 ml complete hair follicle culture medium. The human HFs were exposed to 3.6 kV of NTAPP for 1 min per day 7 consecutive days. Under a stereomicroscope dermal papillae were separated from individual hair follicles using 31G fine needles. Total RNA from mechanically isolated DPs (15-20 pooled DPs per measurement) was isolated and the expressions of selected genes were measured using quantitative RT-PCR. *P<0.05; **P<0.01.

Supplementary Figure 6. Changes in Ki-67+ hair matrix cells after NTAPP exposure in organ culture model.

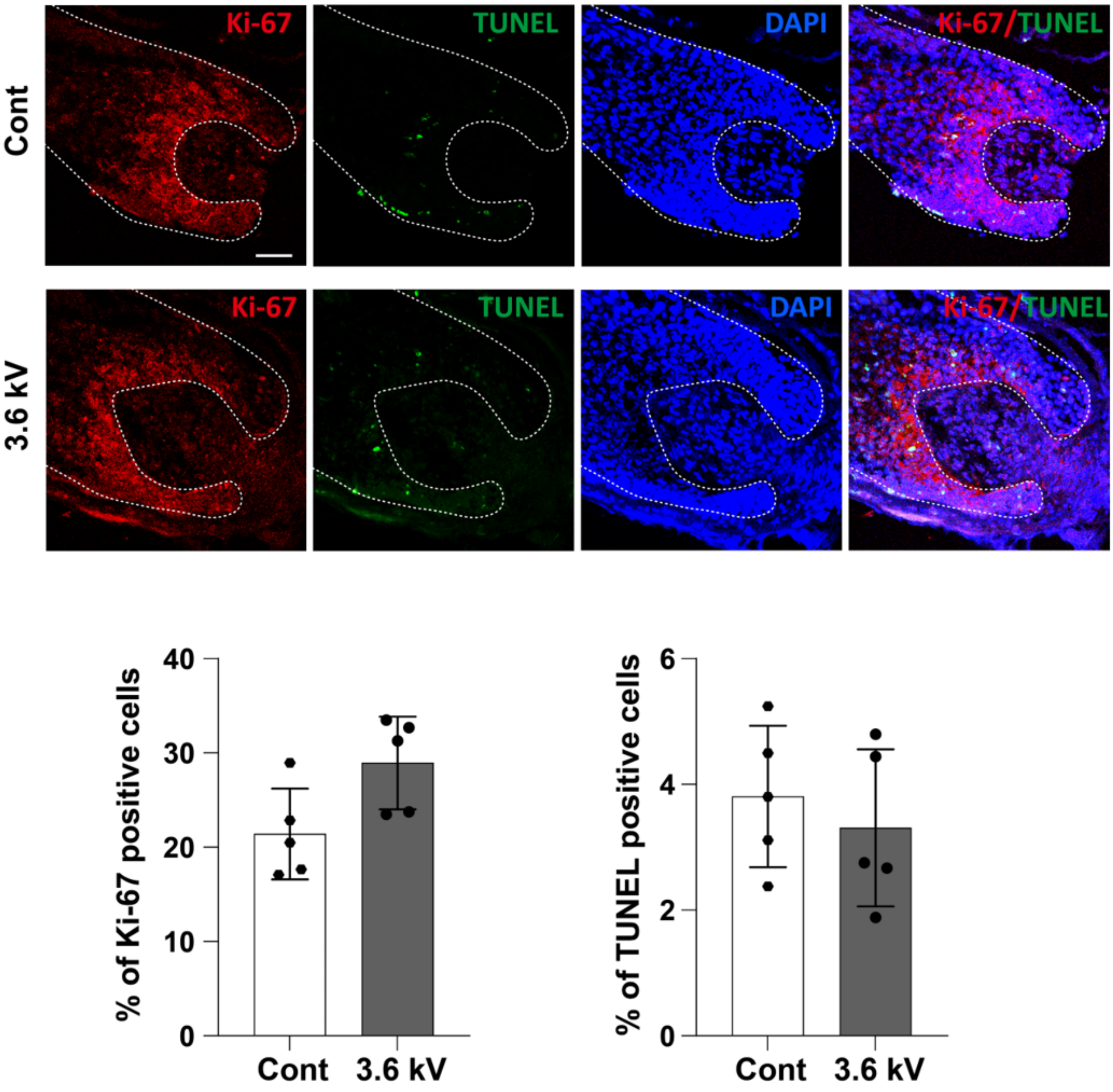

Isolated human hair follicles (HFs) were exposed to 3.6 kV of NTAPP for 1 min per day 7 consecutive days. The HFs were embedded in OCT compound (Tissue Tek, Torranc, CA, USA) at -80°C and then sectioned to 5 µm at -20°C. The sections were fixed and permeabilized then stained with anti-Ki-67 antibody and TUNEL assay kit. The ratio of Ki-67+ cells or TUNEL+ cells among DAPI+ matrix keratinocytes were quantified (n=5 replicates).

Supplementary Figure 7. Expression of β-catenin in dermal papilla after NTAPP exposure in animal model.

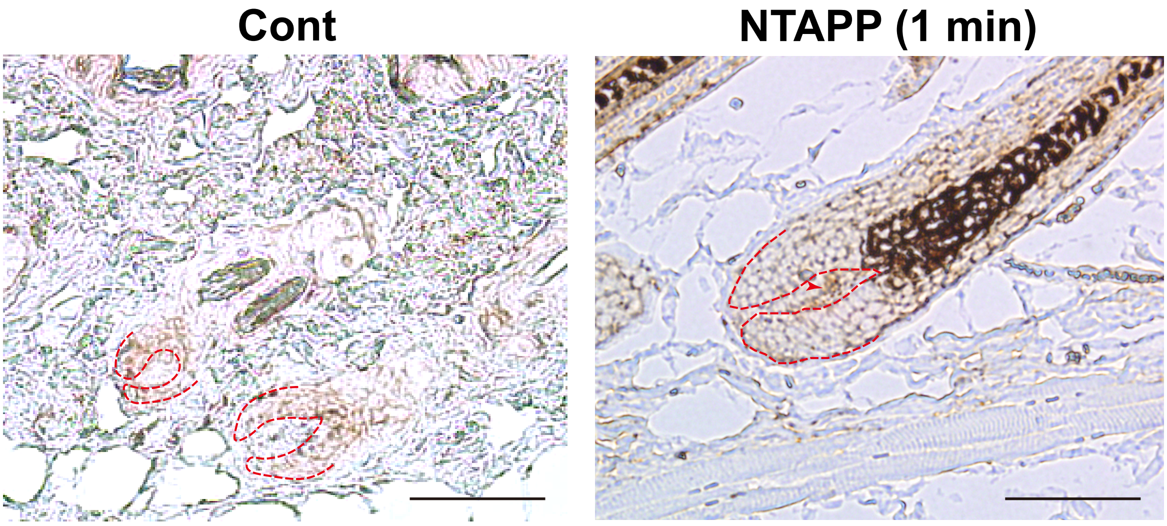

β-catenin expression in hair follicle was examined by immunohistochemistry. Images shown are representative of at least three replicates in 5-μm-thick paraffin section from dorsal skin from NTAPP-treated and control mice at day 16. Dotted lines highlight borders between matrix keratinocytes and dermal papilla and red arrow indicates positive staining for β-catenin. Scale bar = 100 μm.

Supplementary Figure 8. Scheme of a customized NTAPP-generating device.

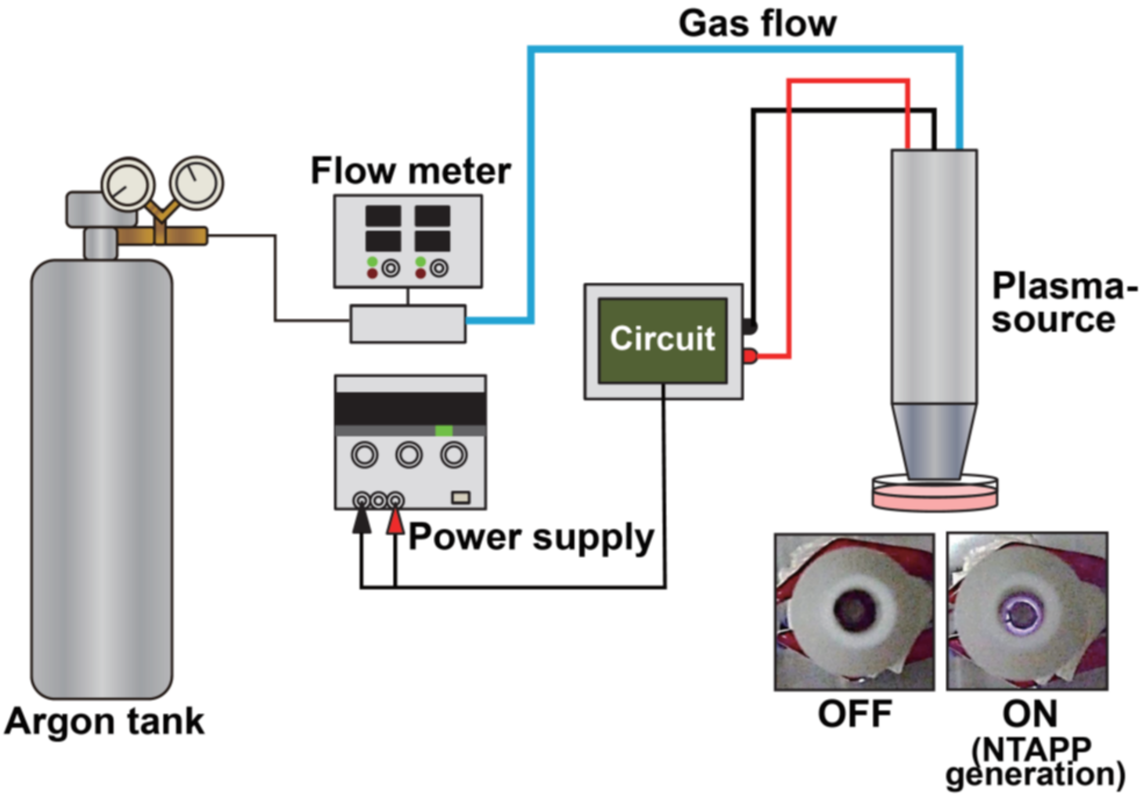

Scheme of a customized, argon-based, NTAPP-generating device.

Table S1. The list of RT-PCR primers used in this study

Gene		Sequence (5' -> 3')
AMER3	Forward	TTCCACATTCGGAGAAACAAGAC
	Reverse	CGAGTCGAAGCTCTGGAGT
NRARP	Forward	TCAACGTGAACTCGTTCGGG
	Reverse	ACTTCGCCTTGGTGATGAGAT
LRG1	Forward	GGACACCCTGGTATTGAAAGAAA
	Reverse	TAGCCGTTCTAATTGCAGCGG
VCAN	Forward	AGGTGGTCTACTTGGGGTGA
	Reverse	CACTTACGTTTAAAGCAGTAGGCA
ALPL	Forward	TCACTCTCCGAGATGGTGGT
	Reverse	TTTCCTTCATGGTGCCCGT
c-Myc	Forward	AATAGAGCTGCTTCGCCTAGA
	Reverse	GAGGTGGTTCATACTGAGCAAG
CCND1	Forward	GCTGCGAAGTGGAAACCATC
	Reverse	CCTCCTTCTGCACACATTTGAA
LEF1	Forward	TGCCAAATATGAATAACGACCCA
	Reverse	GAGAAAAGTGCTCGTCACTGT
TCF4	Forward	CAAGCACTGCCGACTACAATA
	Reverse	CCAGGCTGATTCATCCCACTG
GAPDH	Forward	TGGAAATCCCATCACCATCTTC
	Reverse	CGCCCCACTTGATTTTGG
WNT3A	Forward	GTGGGGTTTTATGGTGGATG
	Reverse	ACCCACAGGAAGGAGCCTAT
WNT5A	Forward	TCCACCTTCCTCTTCACACTGA
	Reverse	CGTGGCCAGCATCACATC
WNT10A	Forward	CTGAACCCCACCACTCACTT
	Reverse	GGCTTGTTTTAGGGTGTGGA
WNT10B	Forward	GGTGCTGCTATGTGCTGTGT
	Reverse	ATCAGAGCAAAGGGCTGAAA
FAS	Forward	AGATTGTGTGATGAAGGACATGG
	Reverse	TGTTGCTGGTGAGTGTGCATT
ACIN1	Forward	GCTGATGACTCTCGCATCTCT
	Reverse	CTGAGTGACTGTCCGGCATA

CASP4	Forward	CATAGAACGACTGTCCAT
	Reverse	TGCTCCTTGAAGTTGATT
BCL2	Forward	GGTGGGGTCATGTGTGTGG
	Reverse	CGGTTCAGGTACTCAGTCATCC
CTNNB1	Forward	CATCTACACAGTTTGATGCTGCT
	Reverse	GCAGTTTTGTCAGTTCAGGGA

Original gels/blots for Figure 1c, e.

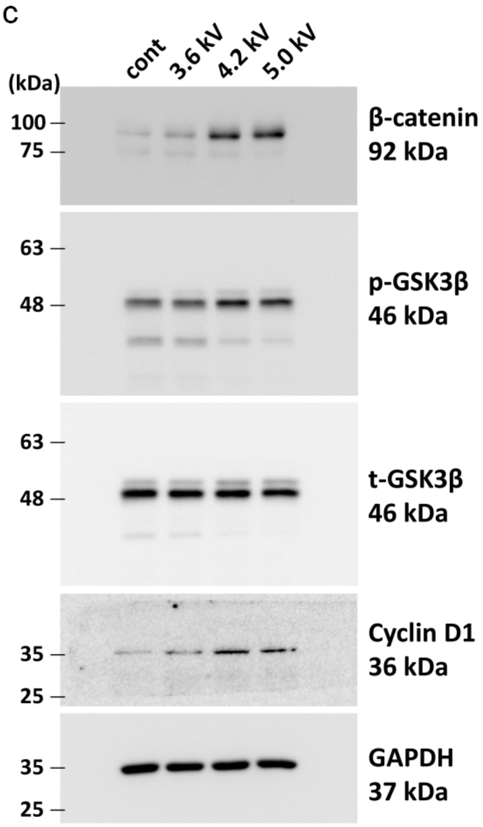

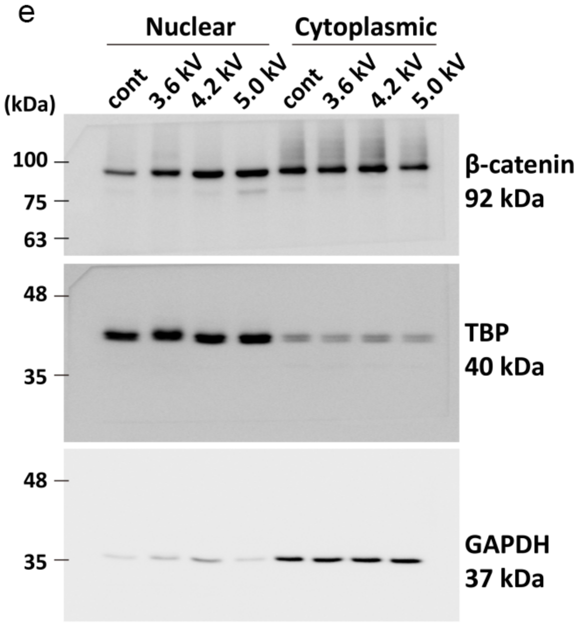

Original gels/blots for Figure 2a, b

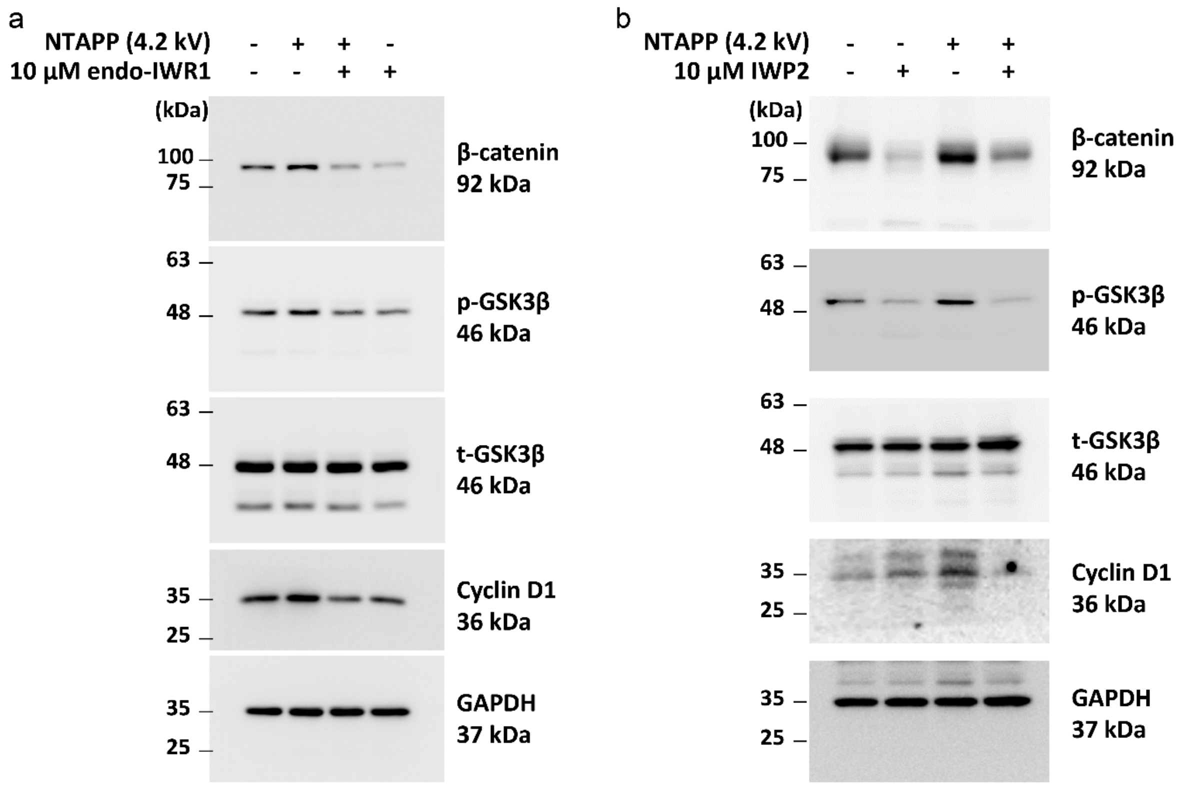

Original gels/blots for Figure 4g.

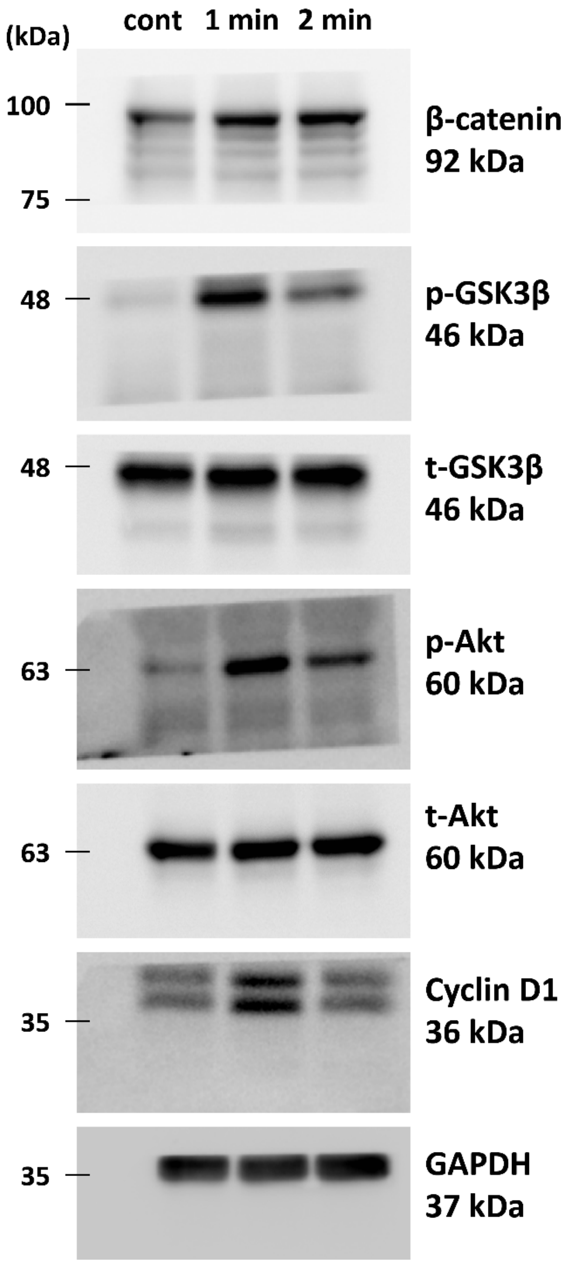

Original gels/blots for Supplementary Figure 4a,c

a

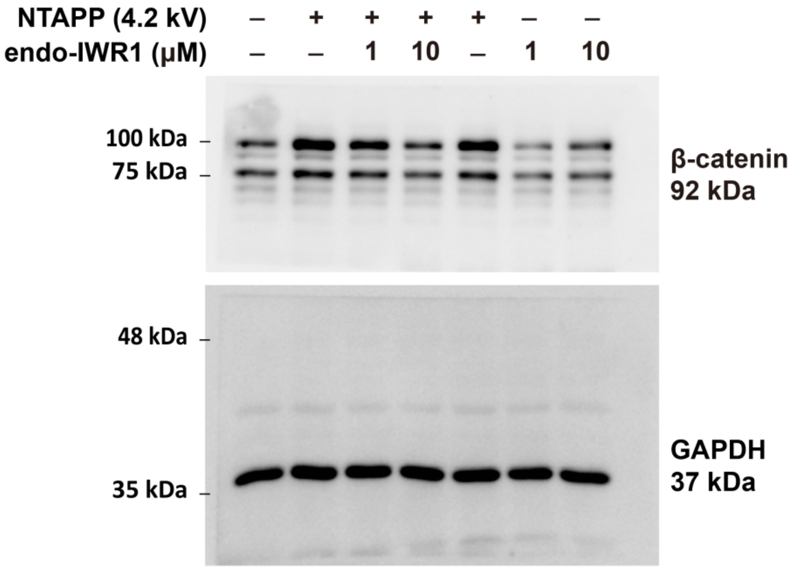

c

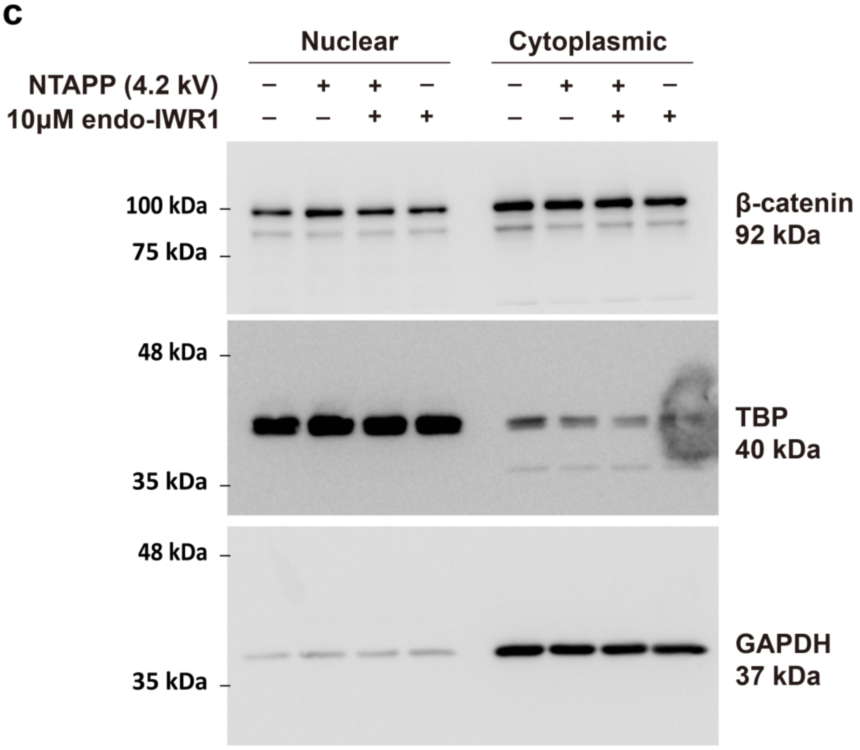

Supplementary Information for MATERIALS AND METHODS

Cell viability, apoptosis, and proliferation assay

After exposure to NTAPP, cell viability was determined by adding 1 ml of 5 mg/ml 3-(4,5-dimethylthiazol-2-yl)-2,5-diphenyl-2H-tetrazolium bromide (MTT; Sigma–Aldrich, St. Louis, MO, USA) in PBS to cells in a culture dish, which was further incubated for 3 h. The supernatant was removed and replaced in 1 ml dimethyl sulfoxide (DMSO; Sigma-Aldrich, St. Louis, MI, USA) to dissolve formazan. The optical density at 570 nm was determined using an ELISA reader. The cell viability rates were calculated from the optical density (OD) readings and are represented as percentages of the control value (untreated cells).

Apoptotic cells were measured using FITC Annexin V Apoptosis Detection kit (BD Biosciences, San Diego, CA, USA) according to the manufacturer's instructions. Briefly, after 18-24 h of incubation, the NTAPP-exposed cells were harvested with trypsin (Lonza, Alpharetta, GA, USA) and washed with phosphate-buffered saline (PBS). Then, cells were stained with FITC-Annexin V and propidium iodide (PI) at room temperature for 15 min avoidingt. FITC-Annexin V and PI assay was performed to discriminate between viable, early apoptotic, late apoptotic and necrotic cells, Data acquisition and analysis were conducted on a flow cytometer (BC FACS LSR II SORP system).

For Ki-67 labeling, NTAPP-exposed hDP cells were incubated in a 35-mm confocal dish (SPL, Korea). The cells were fixed with methanol at room temperature for 5 min. Following the washing with PBS, the cells were treated with 0.1% TritonX-100 (Sigma) for 15 min at RT and incubated with mouse monoclonal anti-human Ki-67 antigen (clone MIB-1, DAKO, 1:100) for 18-24 h at 4 °C. Immnuocytochemistry for Ki-67 was carried out detection via Dako Real EnVision Dectection System Peroxidas/DAB+, Rabbit/Mouse (DAKO). Only distinct nuclear

staining of cells was used for scoring via the light microscope, which was determined semi-quantitatively as nil (no immunostaining), low (10% or less immune-positivity) or high (>10% immune-reactive cells) respectively.

Western blot analysis

Cells and tissue were lysed in RIPA buffer (GenDEPOT, Houston, TX, USA). Protein samples were resolved on SDS-PAGE and transferred onto a nitrocellulose membrane (GE Healthcare Life science, Germany). After blocking with 2.5% skim milk (Becton-Dickinson, CA, USA), the membranes were incubated with primary rabbit antibodies: anti-β-catenin (1:1000), anti-pGSK3β (Ser9, 1:1000), anti- tGSK3β (1:1000), anti-cyclin D1 (1:1000), anti-p-Akt (1:1000), anti-tAkt (1:1000) (all from Cell Signaling Technology, Danvers, MA, USA), or anti-GAPDH (1:5000; Santa Cruz Biotechnology, Dallas, TX, USA). Finally, the membranes were then incubated with the corresponding secondary antibody, peroxidase-conjugated affinity-purified goat anti-rabbit IgG or goat anti-rabbit IgG secondary antibody (1:10000; GenDEPOT). For detecting of phosphorylated proteins, the membranes were stripped with stripping buffer (Thermo Scientific, USA) according to manufacturer's instructions; the levels of total protein were measured by conducting the second hybridization. The protein bands were detected using an ECL Plus kit (Millipore Corporation, Billerica, MA, USA).

RNA isolation and quantitative RT-PCR

Total RNA from cells was isolated with Trizol reagent (Qiagen GmbH, Hilden, Germany). After synthesis of first-strand cDNA (SuperScript III Reverse Transcriptase; Invitrogen), a quantitative SYBR Green RT-PCR kit (Applied Biosystems, Warrington, UK) was used with a Step One Plus RT-PCR System (Applied Biosystems). The list of primers used during experiments is shown in

Supplementary Table 1. The PCR was conducted under the following conditions: Denaturation at 95°C for 15 sec, 40 amplification cycles of annealing at 60°C for 30 sec, and extension at 72°C for 30 sec. All samples were run in triplicate, and relative gene expression was determined using the $2^{-\Delta\Delta Ct}$ method, with *GAPDH* as a normalization standard.

Immunohistochemical staining

The skin samples of NTAPP-exposed mouse dorsal skin at Day 16 were fixed with formalin and embedded in paraffin and then sectioned into 5 μm-thick. The tissue sections were deparaffinized and rehydrated via sequential washing with xylene, graded ethanol and PBS, and then performed antigen retrieval in 1X citrate buffer (sigma) using the microwave heating method. After washing with PBS, the sections were treated with normal goat serum blocking solution and then incubated with β-catenin primary antibody (1:40, cell signaling) overnight at 4’C. Antibody binding was detected using Dako Real EnVision Dectection System Peroxidas/DAB+, Rabbit/Mouse (DAKO) for immunohistochemistry. The slides were examined and images were taken using an Olympus 1X73 fluorescence microscope.
